# Identifying risk and prognostic factors for synchronous liver metastasis in small bowel adenocarcinoma: a predictive analysis using the SEER database

**DOI:** 10.3389/fsurg.2024.1437124

**Published:** 2024-07-29

**Authors:** Duogang Xu, Yulei He, Changkang Liao, Jing Tan

**Affiliations:** ^1^ Department of General Surgery, Yan'an Hospital Affiliated to Kunming Medical University, Kunming, China; ^2^ Key Laboratory of Tumor Immunological Prevention and Treatment of Yunnan Province, Kunming, China; ^3^ The First School of Clinical Medicine, Yunnan University of Chinese Medicine, Kunming, China

**Keywords:** small bowel adenocarcinoma, liver metastasis, Surveillance, Epidemiology, and End Results (SEER), predictive nomogram, cancer-specific survival

## Abstract

**Background:**

Small bowel adenocarcinoma (SBA) is a rare gastrointestinal malignancy with an increasing incidence and a high propensity for liver metastasis (LM). This study aimed to investigate the risk factors for synchronous LM and prognostic factors in patients with LM.

**Methods:**

Utilizing the Surveillance, Epidemiology, and End Results (SEER) database, this study analyzed data from 2,064 patients diagnosed with SBA between 2010 and 2020. Logistic regression was used to determine risk factors for synchronous LM. A nomogram was developed to predict the risk of LM in SBA patients, and its predictive performance was assessed through receiver operating characteristic (ROC) curves and calibration curves. Kaplan-Meier and Cox regression analyses were conducted to evaluate survival outcomes for SBA patients with LM.

**Results:**

Synchronous LM was present in 13.4% of SBA patients (*n* = 276). Six independent predictive factors for LM were identified, including tumor location, T stage, N stage, surgical intervention, retrieval of regional lymph nodes (RORLN), and chemotherapy. The nomogram demonstrated good discriminative ability, with an area under the curve (AUC) of 83.8%. Patients with LM had significantly lower survival rates than those without LM (*P* < 0.001). Survival analysis revealed that advanced age, tumor location in the duodenum, surgery, RORLN and chemotherapy were associated with cancer-specific survival (CSS) in patients with LM originating from SBA.

**Conclusions:**

This study highlights the significant impact of LM on the survival of SBA patients and identifies key risk factors for its occurrence. The developed nomogram aids in targeted screening and personalized treatment planning.

## 1 Introduction

Primary small bowel tumors are rare neoplasms within the gastrointestinal tract, accounting for approximately 5% of all gastrointestinal tumors, with malignant forms comprising only 1%–2% of these cases (1). Adenocarcinomas of the small bowel represent approximately 40% of all primary small bowel tumors (2) and frequently metastasize to the liver, followed by the lungs and bones (3). In contrast to that of colorectal cancer (CRC), the incidence of small bowel adenocarcinoma (SBA) has been increasing annually in the United States and Europe, with thousands of new cases reported each year (4, 5).

The presence of liver metastasis (LM) is a significant predictor of poor prognosis in SBA patients, likely due to advanced disease progression leading to cachexia and impaired liver function (6, 7). Early identification of high-risk individuals prone to synchronous LM could enable clinicians to conduct targeted screening and personalized treatment, potentially improving survival rates. Therefore, identifying the risk factors associated with the occurrence and prognosis of synchronous LM in SBA patients is meaningful (8, 9).

Due to the rarity of LM from SBA, few studies have reported on the prognosis of this patient population. Moreover, because the clinical symptoms are not prominent, most patients are diagnosed at an advanced stage (10, 11). An earlier multicenter study of chemotherapy in patients with advanced SBA in France suggested that World Health Organization performance status (PS), elevated serum carcinoembryonic antigen (CEA) and carbohydrate antigen 19-9 (CA 19-9) were independent risk factors for overall survival (OS) (12). Another multicenter study evaluated prognostic factors in patients with surgically resected metastatic SBA suggesting that poor differentiation, borderline infiltration, and lymphatic infiltration of the primary tumor were associated with reduced OS (13).

Artificial intelligence (AI) and deep learning (DL) are currently incorporated into all diagnostic processes for tumors such as CRC and SBA, ranging from histopathological images for classification and endoscopic tumor identification to radiological diagnosis via CT scanning and CT colonoscopy, as well as further serological screening tests (14). Advances enabled by DL algorithms have the potential to improve the accuracy and effectiveness of gastrointestinal tumor detection (15). Additionally, an increasing number of studies are employing nomograms as graphical predictive models, which allow survival prediction points to be calculated based on predictors, guiding targeted treatment plans (16). Consequently, there is a need to establish a quantitative predictive model for assessing the risk of LM in patients with SBA to facilitate timely and economical screening for LM. Furthermore, prognostic models can aid clinicians in providing targeted therapeutic strategies for patients.

In this study, we utilized the Surveillance, Epidemiology, and End Results (SEER) database to investigate the incidence and predictive factors of synchronous LM in SBA patients and developed a corresponding nomogram. We also explored prognostic factors related to the survival of SBA patients with LM, aiming to contribute to the optimization of diagnostic and therapeutic strategies for LM in SBA patients. This article is presented following the TRIPOD reporting checklist (https://dx.doi.org/10.21037/apm-21-600).

## 2 Materials and methods

### 2.1 Patient selection

The data for this population-based study were sourced from the SEER database of the National Cancer Institute. For approximately 34.6% of the U.S. population, the SEER database collates cancer incidence data from 18 registries and includes detailed patient demographic, treatment, and survival information (17). We accessed the data using SEER*Stat software version 8.3.6. The study cohort comprised patients diagnosed with SBA between 2010 and 2020, totaling 6,641 individuals. Patients were identified using the SEER variables “Site Recode ICD-O-3/WHO 2008 classification” (small bowel) and “Histology Recode—Broad Group” (histology codes: 8140-8389). Survival data were extracted using the codes “SEER Cause-Specific Death Classification” and “Survival Months”. Cancer-specific survival (CSS) was defined as the interval between the diagnosis of SBA and death from SBA.

**Figure 1:**
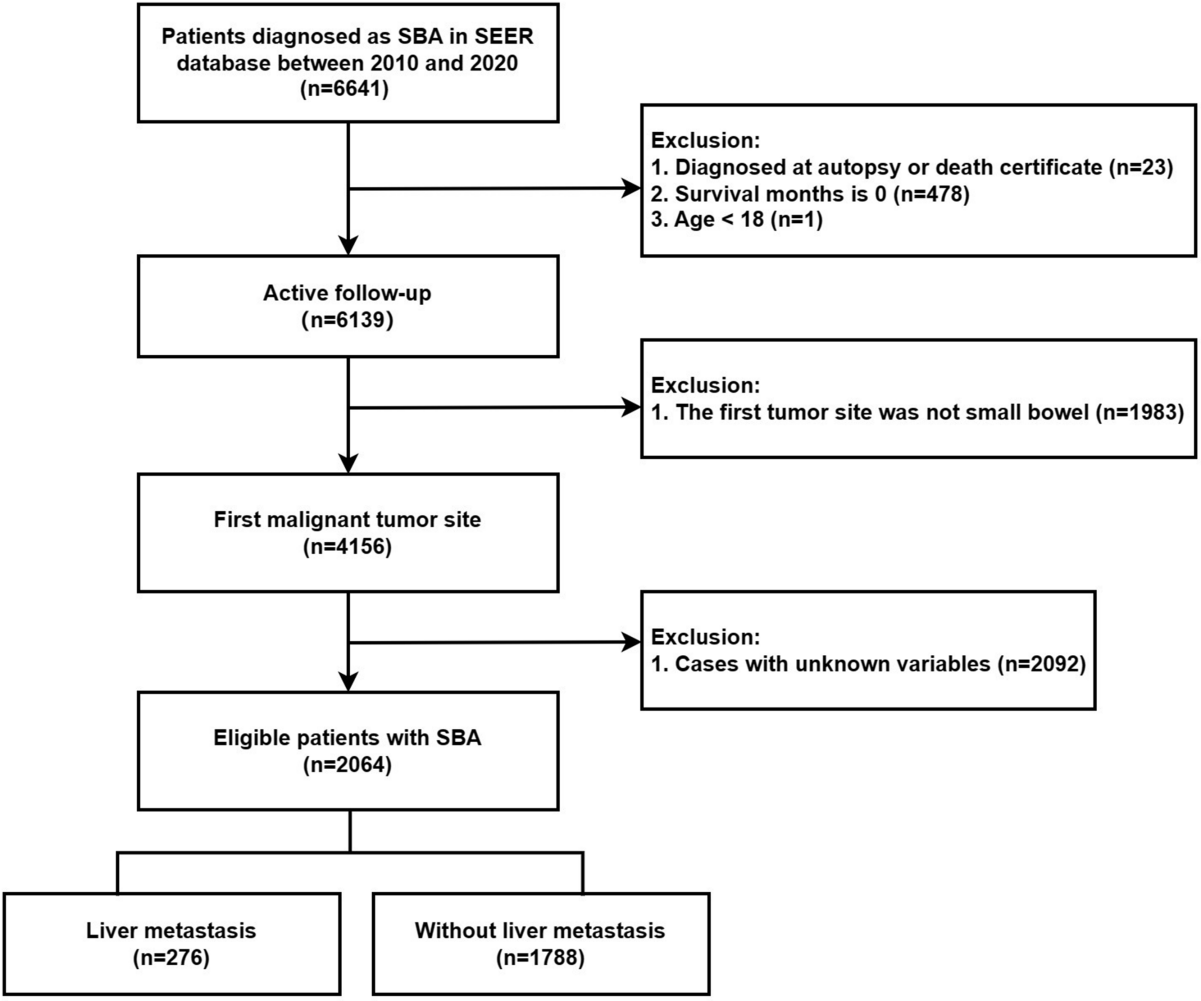
Flow chart for the selection of eligible patients with adenocarcinoma of the small bowel.

The exclusion criteria were as follows: (1) patients confirmed by death certificate or autopsy only; (2) patients with a survival time of 0 months; (3) patients younger than 18 years; (4) patients whose first primary tumor was not SBA; and (5) patients whose clinical-pathological information was incomplete. The exclusion process is illustrated in Figure 1. Ultimately, 2,064 patients were included in the study cohort, consisting of 276 patients with synchronous LM and 1,788 without LM. Patients with SBA were randomly assigned to the training and validation groups in an 8:2 ratio. A binary logistic regression analysis was conducted on the training group to investigate the risk factors associated with synchronous LM. Subsequently, the 276 patients with SBA LM who met the inclusion criteria were analyzed using univariate and multivariate Cox regression analyses to explore factors influencing their prognosis.

### 2.2 Study variables and endpoints

Data concerning variables such as sex, age at diagnosis, marital status, race, tumor location, histologic grade, tumor stage (T), lymph node involvement (N), surgical intervention, retrieval of regional lymph nodes (RORLN), radiation therapy, chemotherapy, and the presence of synchronous LM were extracted from the SEER database. The facial categories were defined as White, Black, and Other. The tumor sites were classified as duodenum, jejunum, ileum, or other/unspecified. Tumors were graded based on differentiation as Grade I (well-differentiated), Grade II (moderately differentiated), Grade III (poorly differentiated), or Grade IV (undifferentiated). Tumor staging was specified as T1-2, T3-4, or Tx, with N staging described as N0, N1-N2, or Nx, respectively. Surgical treatments were categorized as none, radical, or palliative. Radical surgery was defined as the concurrent resection of the primary and metastatic tumors within the same procedure, whereas palliative surgery involved various degrees of resection of the primary tumor, with or without metastasis. The number of regional lymph nodes dissected was categorized as 0, 1-3, or ≥4. In this study, CSS was utilized as the prognostic endpoint for patients with SBA who developed LM. This endpoint focuses on survival specifically attributed to SBA, excluding deaths from other causes.

### 2.3 Statistical analysis

The clinical and pathological characteristics of the SBA cohorts with and without LM were compared using the chi-square test to determine baseline differences. Survival analysis was conducted using the Kaplan‒Meier method, and differences in survival between the two groups were assessed using the log-rank test. Multicollinearity among independent variables was addressed using a bidirectional stepwise selection approach in logistic and Cox regression models. Univariate and multivariate logistic regression analyses were employed to evaluate all variables to identify independent prognostic factors for developing LM in SBA patients. A nomogram was developed based on these identified factors. The predictive accuracy of the nomogram was assessed using the area under the receiver operating characteristic (ROC) curve. Calibration curves were generated through 1,000 bootstrap resamplings to evaluate the calibration of the nomogram. Furthermore, independent prognostic factors were identified using a multivariate Cox proportional hazards model, and the associated hazard ratio (HR) and 95% confidence interval (CI) were calculated. A two-sided *P* value less than 0.05 was considered to indicate statistical significance. All analyses were conducted using R software (version 4.3.2; http://www.r-project.org).

## 3 Results

### 3.1 Clinical and pathological characteristics of patients

This study included 2,064 patients from the SEER database who met the eligibility criteria. The demographic and clinicopathological characteristics of the overall cohort and subgroups are summarized in Table 1. Predominantly, the cohort consisted of males (1,132 patients, 54.8%), and individuals aged ≥70 years composed the largest age group (744 patients, 36%). The duodenum was the most common site of SBA (1,082 patients, 52.4%). Surgical intervention was performed in 1,651 patients (80%), and chemotherapy was administered to 1,103 patients (53.4%). Additionally, a significant number of patients (1,278, 61.9%) had ≥4 regional lymph nodes dissected. The proportion of patients with no LM vs. LM was 86.6% vs. 13.4%, respectively. Significant differences were observed between patients with and without LM regarding tumor location, histologic grade, T stage, N stage, surgical treatment, RORLN, and chemotherapy usage. Specifically, for patients with LM from SBA, the majority were older males; the duodenum was the most common primary tumor site (64.1%); most tumors were moderately differentiated (47.1%); primary tumors tended to be larger (58.3%); lymph node metastasis occurred in 50% of patients; a majority of patients possibly missed surgical opportunities (62.6%); fewer regional lymph nodes were dissected during surgery (67.7%); and most patients with metastasis underwent chemotherapy (69.2%).

**Table 1:** Patient baseline characteristics.

Variables	Total (*n* = 2,064)	Without LM^a^ (*n* = 1,788)	With LM (*n* = 276)	*P* value
Gender, *n* (%)				0.211
Male	1,132 (54.84)	971 (54.31)	161 (58.33)	
Female	932 (45.16)	817 (45.69)	115 (41.67)	
Age, *n* (%)				0.514
≤50	310 (15.02)	266 (14.88)	44 (15.94)	
51–60	445 (21.56)	390 (21.81)	55 (19.93)	
61–70	565 (27.37)	485 (27.13)	80 (28.99)	
≥70	744 (36.05)	647 (36.19)	97 (35.14)	
Marital status, *n* (%)				0.206
Married	1,253 (60.71)	1,095 (61.24)	158 (57.25)	
Unmarried	811 (39.29)	693 (38.76)	118 (42.75)	
Race, *n* (%)				0.083
White	1,531 (74.18)	1,340 (74.94)	191 (69.20)	
Black	374 (18.12)	311 (17.39)	63 (22.83)	
Other	159 (7.7)	137 (7.66)	22 (7.97)	
Tumor site, *n* (%)				<0.001
Duodenum	1,082 (52.42)	905 (50.62)	177 (64.13)	
Jejunum	374 (18.12)	329 (18.40)	45 (16.30)	
Ileum	345 (16.72)	319 (17.84)	26 (9.42)	
Other/NOS^a^	263 (12.74)	235 (13.14)	28 (10.14)	
Histological grade, *n* (%)				0.004
I	204 (9.88)	189 (10.57)	15 (5.43)	
II	1,054 (51.07)	924 (51.68)	130 (47.10)	
III	777 (37.65)	651 (36.41)	126 (45.65)	
IV	29 (1.41)	24 (1.34)	5 (1.81)	
T stage, *n* (%)				<0.001
T1-T2	343 (16.62)	297 (16.61)	46 (16.67)	
T3-T4	1,602 (77.62)	1,441 (80.59)	161 (58.33)	
Tx	119 (5.77)	50 (2.80)	69 (25.00)	
N stage, *n* (%)				<0.001
N0	1,023 (49.56)	914 (51.12)	109 (39.49)	
N1-N2	988 (47.87)	850 (47.54)	138 (50.00)	
Nx	53 (2.57)	24 (1.34)	29 (10.51)	
Surgery, *n* (%)				<0.001
No	413 (20.01)	240 (13.42)	173 (62.68)	
Palliative surgery	1,220 (59.11)	1,134 (63.42)	86 (31.16)	
Radical surgery	431 (20.88)	414 (23.15)	17 (6.16)	
RORLN^a^, *n* (%)				<0.001
0	594 (28.78)	407 (22.76)	187 (67.75)	
1–3	192 (9.30)	173 (9.68)	19 (6.88)	
≥4	1,278 (61.92)	1,208 (67.56)	70 (25.36)	
Radiation, *n* (%)				0.880
No	1,882 (91.18)	1,631 (91.22)	251 (90.94)	
Yes	182 (8.82)	157 (8.78)	25 (9.06)	
Chemotherapy, *n* (%)				<0.001
No	961 (46.56)	876 (48.99)	85 (30.80)	
Yes	1,103 (53.44)	912 (51.01)	191 (69.20)	

^a^ LM, liver metastasis; NOS, not otherwise specified; RORLN, retrieval of regional lymph nodes.

### 3.2 Survival analysis for LM patients

**Figure 2:**
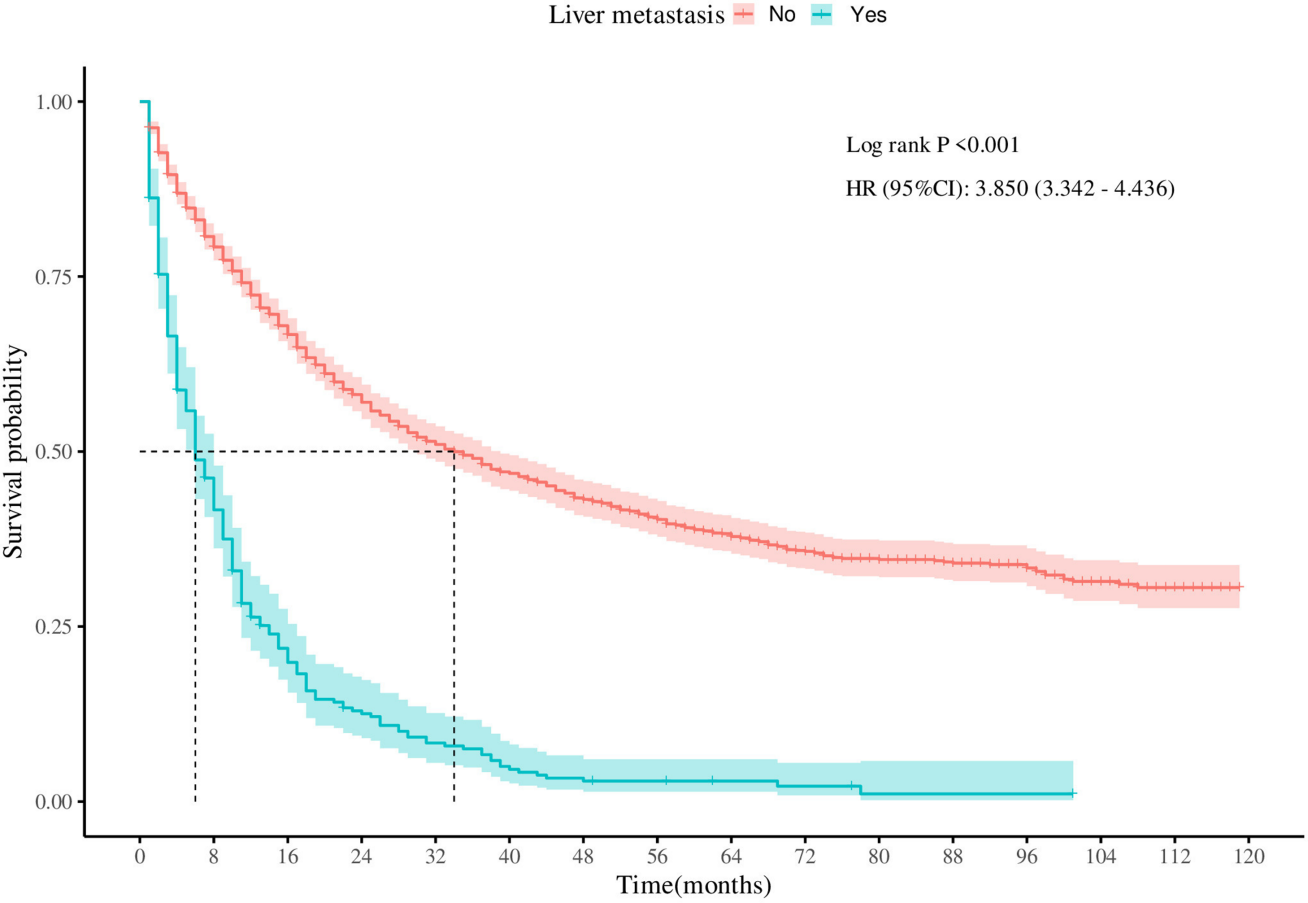
Survival curves for cancer-specific survival in patients with adenocarcinoma of the small bowel with or without liver metastases.

To assess the survival outcomes of patients with LM from SBA, Kaplan‒Meier survival analysis was performed on the entire cohort. As illustrated in Figure 2, there was a statistically significant difference in CSS between patients with and without LM (*P* < 0.001). Patients without LM had a median survival time of 34 months. In contrast, patients with LM exhibited a poorer prognosis, with a median survival time of only 6 months.

### 3.3 Risk factors for LM in SBA patients

**Figure 3:**
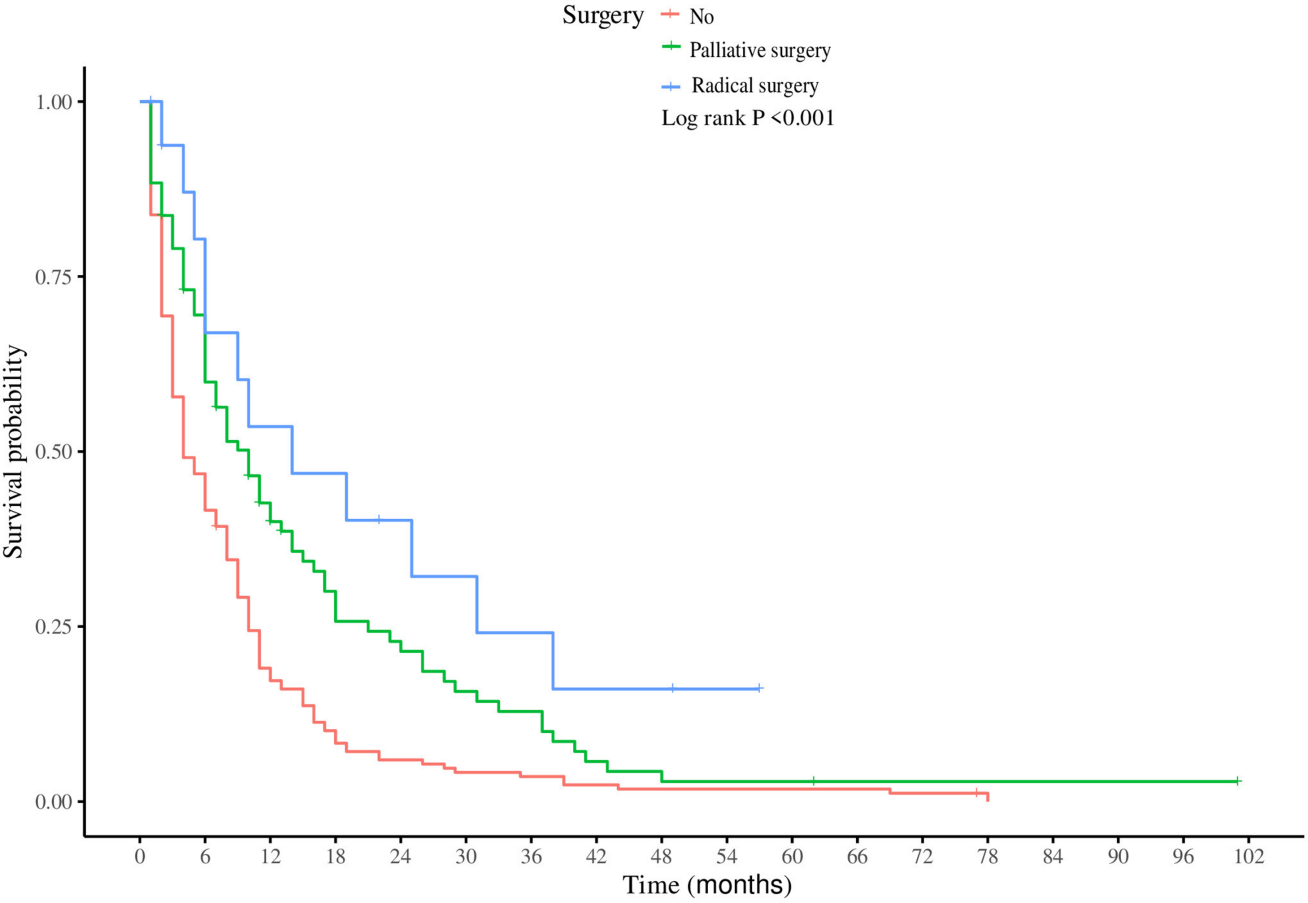
Nomogram for predicting the risk of liver metastases in patients with adenocarcinoma of the small bowel.

In the analysis of risk factors for LM among SBA patients, univariate logistic regression was initially used to screen potential risk factors. To determine whether these factors could act as independent risk factors, multivariate logistic regression was subsequently employed to confirm the independent risk factors and quantify their influence on the likelihood of LM, represented by odds ratios (ORs). Preliminary risk factors (with a *P* value <0.05 in univariate analysis) were included in the multivariate logistic regression model for further analysis. Six variables were identified as independent predictors of LM in SBA patients: primary tumor location, T stage, N stage, surgical intervention, RORLN, and chemotherapy (as shown in Table 2, all with *P* values <0.05 in the multivariate analysis). A nomogram was created using these six statistically significant variables (Figure 3). By adding the scores associated with each variable and projecting the total score onto a bottom scale, the probability of LM occurring in SBA patients can be easily calculated. The nomogram revealed that whether surgery was performed had the most substantial impact on the risk of LM in SBA patients.

**Table 2:** Univariate and multivariate logistic regression analyses of risk factors for developing liver metastases from adenocarcinoma of the small bowel.

Variables	Univariate OR (95% CI)	*P* value	Multivariate OR (95%CI)	*P* value
Gender				
Male	Reference			
Female	0.89 (0.67–1.18)	0.434		
Age				
≤50	Reference			
51–60	0.94 (0.59–1.51)	0.809		
61–70	1.00 (0.64–1.56)	0.998		
≥70	0.93 (0.61–1.42)	0.733		
Marital status				
Married	Reference			
Unmarried	1.11 (0.84–1.47)	0.463		
Race				
White	Reference			
Black	1.35 (0.96–1.90)	0.083		
Other	1.11 (0.66–1.85)	0.691		
Tumor site				
Duodenum	Reference		Reference	
Jejunum	1.18 (1.11–1.53)	0.136	1.83 (1.10–3.04)	0.020
Ileum	1.42 (1.28–1.63)	<0.001	1.76 (1.00–3.12)	0.051
Other/NOS^a^	0.67 (0.44–0.91)	0.011	1.05 (0.57–1.94)	0.871
Histological grade				
I	Reference		Reference	
II	1.62 (0.90–2.90)	0.105	1.56 (0.80–3.04)	0.193
III	2.17 (1.21–3.91)	0.010	1.44 (0.73–2.83)	0.289
IV	2.19 (0.66–7.29)	0.203	1.67 (0.41–6.77)	0.470
T stage				
T1-T2	Reference		Reference	
T3-T4	0.67 (0.45–0.99)	0.043	1.07 (0.66–1.73)	0.790
Tx	8.26 (4.88–14.01)	<0.001	2.39 (1.32–4.31)	0.004
N stage				
N0	Reference		Reference	
N1-N2	1.46 (1.08–1.96)	0.013	1.97 (1.36–2.86)	<0.001
Nx	10.11 (5.29–19.33)	<0.001	2.29 (1.11–4.73)	0.026
Surgery				
No	Reference		Reference	
Palliative surgery	0.10 (0.07–0.14)	<0.001	0.16 (0.08–0.30)	<0.001
Radical surgery	0.06 (0.04–0.10)	<0.001	0.11 (0.05–0.25)	<0.001
RORLN^a^				
0	Reference		Reference	
1–3	0.19 (0.11–0.35)	<0.001	0.64 (0.30–1.37)	0.251
≥4	0.13 (0.09–0.17)	<0.001	0.44 (0.24–0.83)	0.011
Radiation				
No	Reference			
Yes	1.11 (0.69–1.79)	0.661		
Chemotherapy				
No	Reference		Reference	
Yes	2.03 (1.51–2.73)	<0.001	1.92 (1.35–2.73)	<0.001

^a^ NOS, not otherwise specified; RORLN, retrieval of regional lymph nodes.

### 3.4 Validation of nomograms

**Figure 4:**
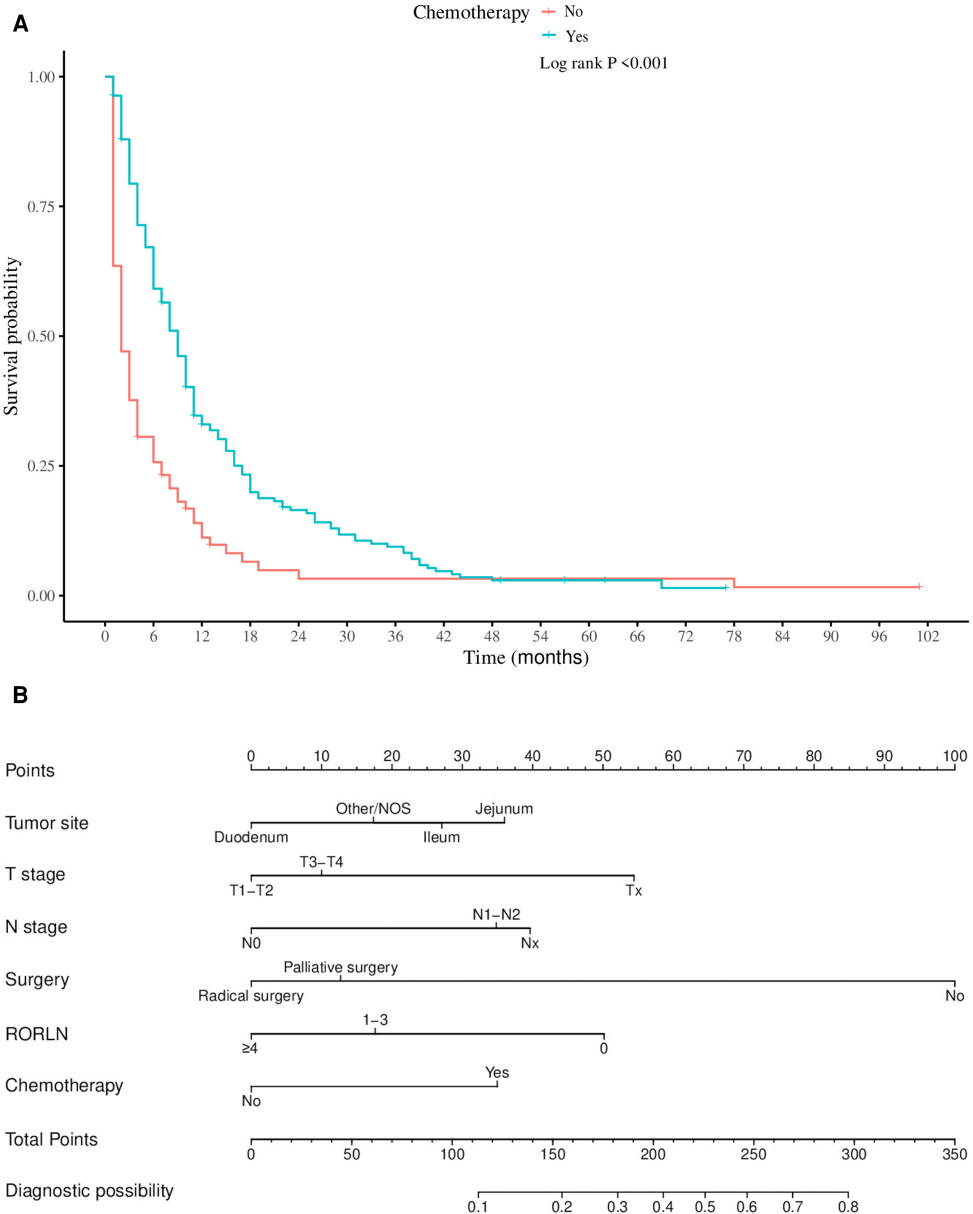
Validation of the nomograms. (**A**) ROC curve. (**B**) Calibration curve.

To evaluate the discriminative ability and calibration of the nomogram developed for predicting LM in SBA patients, various validation methods were employed, including ROC curve and calibration curve analyses. The ROC curve, as shown in Figure 4A, demonstrated good discriminative power. The area under the curve (AUC) for predicting LM was 83.8%, with a 95% CI of 80.1 −86.4%. This indicates a high level of accuracy in the nomogram's predictions relative to the actual outcomes. Furthermore, the nomogram's calibration was assessed using the bootstrap resampling method, with 1,000 replications performed to ensure robustness. The calibration curve, displayed in Figure 4B, showed good agreement between the predicted probabilities and observed outcomes.

### 3.5 Survival analysis for patients with LM from SBA

**Figure 5:**
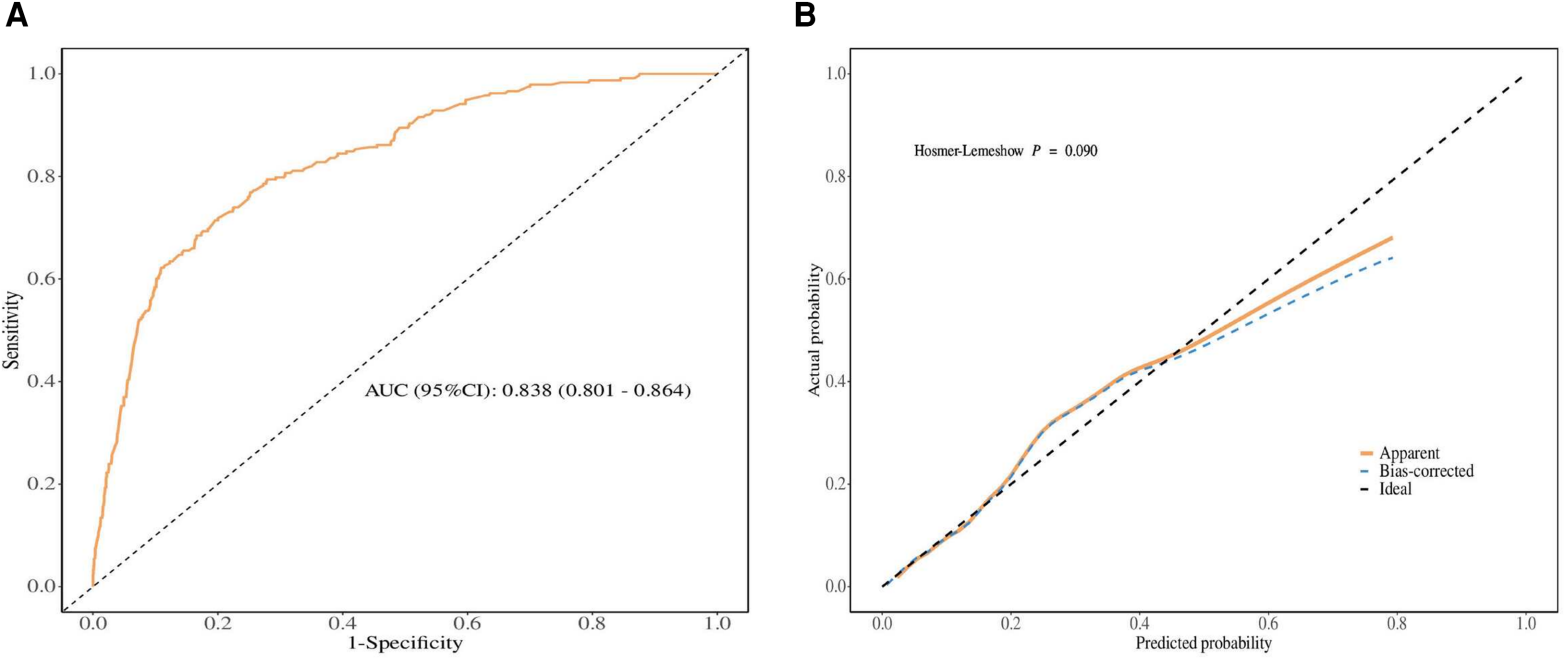
Kaplan-meier survival analysis of surgical and chemotherapy variables in patients with hepatic metastases from small bowel adenocarcinoma. (**A**) surgery (**B**) chemotherapy.

In the subgroup of patients with LM from SBA, both univariate and multivariate Cox regression analyses were performed to identify factors significantly associated with CSS (Table 3). Univariate analysis indicated that older age, primary tumors located in the duodenum, higher T stages, absence of surgical treatment, fewer regional lymph nodes dissected, and lack of chemotherapy were all positively correlated with increased mortality. Multivariate Cox regression analysis revealed several key findings: Patients aged ≥70 years had poorer CSS than did those under 50 years (HR = 1.59, 95% CI: 1.22–1.84, *P* = 0.027). Patients with primary tumors located in the jejunum had a better prognosis than those with tumors located in the duodenum (HR = 0.73, 95% CI: 0.65–0.82; *P* = 0.007). Compared to those who did not undergo surgery, patients who underwent palliative surgery (HR = 0.77, 95% CI: 0.53–0.92, *P* = 0.012) or radical surgery (HR = 0.64, 95% CI: 0.59–0.71, *P* = 0.009) exhibited better survival. Patients with ≥4 regional lymph nodes dissected during surgery had better outcomes (HR = 0.59, 95% CI: 0.33–0.77; *P* = 0.023). Patients who received chemotherapy post metastasis had a lower risk of death than did those who did not receive chemotherapy (HR = 0.46, 95% CI: 0.35–0.61, *P* < 0.001). Furthermore, Kaplan‒Meier survival analysis for the variables of surgery and chemotherapy in patients with SBA-LM was conducted. The survival curves visually demonstrated that patients who underwent radical surgery had the highest survival rates, followed by those who underwent palliative surgery, with the poorest prognosis observed in those who did not undergo any surgery (*P* < 0.001) (Figure 5A). Similarly, patients who received chemotherapy had higher survival rates than did those who did not receive chemotherapy (*P* < 0.001) (Figure 5B).

**Table 3:** Univariate and multifactorial Cox regression analyses of prognostic factors in patients with hepatic metastases from adenocarcinoma of the small bowel.

Variables	Univariate HR (95% CI)	*P* value	Multivariate HR (95%CI)	*P* value
Gender				
Male	Reference			
Female	1.13 (0.88–1.45)	0.321		
Age				
≤50	Reference		Reference	
51–60	1.02 (0.67–1.55)	0.917	0.78 (0.50–1.20)	0.256
61–70	1.26 (0.85–1.86)	0.250	1.15 (0.77–1.72)	0.490
≥70	1.49 (1.02–2.18)	0.010	1.59 (1.22–1.84)	0.027
Marital status				
Married	Reference			
Unmarried	1.08 (0.85–1.39)	0.523		
Race				
White	Reference			
Black	0.91 (0.68–1.22)	0.540		
Other	0.81 (0.51–1.31)	0.395		
Tumor site				
Duodenum	Reference		Reference	
Jejunum	0.57 (0.40–0.81)	0.002	0.73 (0.65–0.82)	0.007
Ileum	0.74 (0.48–1.14)	0.170	1.26 (0.70–2.28)	0.437
Other/NOS^a^	0.86 (0.56–1.30)	0.467	0.97 (0.63–1.50)	0.891
Histological grade				
I	Reference			
II	0.76 (0.43–1.35)	0.351		
III	1.02 (0.58–1.81)	0.946		
IV	1.10 (0.36–3.39)	0.863		
T stage				
T1-T2	Reference		Reference	
T3-T4	1.46 (1.41–1.55)	0.032	1.83 (0.57–1.21)	0.233
Tx	0.89 (0.61–1.29)	0.529	0.80 (0.54–1.17)	0.242
N stage				
N0	Reference			
N1-N2	0.79 (0.61–1.02)	0.076		
Nx	0.82 (0.54–1.24)	0.337		
Surgery				
No	Reference		Reference	
Palliative surgery	0.60 (0.46–0.79)	<0.001	0.77 (0.53–0.92)	0.012
Radical surgery	0.39 (0.22–0.70)	0.002	0.64 (0.59–0.71)	0.009
RORLN^a^				
0	Reference		Reference	
1–3	0.67 (0.41–1.11)	0.122	0.83 (0.45–1.54)	0.356
≥4	0.51 (0.37–0.68)	<0.001	0.59 (0.33–0.77)	0.023
Radiation				
No	Reference			
Yes	1.31 (0.87–1.99)	0.199		
Chemotherapy				
No	Reference		Reference	
Yes	0.48 (0.37–0.63)	<0.001	0.46 (0.35–0.61)	<0.001

^a^ NOS, not otherwise specified; RORLN, retrieval of regional lymph nodes.

## 4 Discussion

Epidemiologically, SBA is relatively rare but has shown a steady increase in annual incidence (18). Our study explored the challenges of predicting and improving outcomes for patients with LM originating from SBA, emphasizing the complexity of managing this rare yet challenging cancer type. Early identification of LM and appropriate therapeutic interventions can significantly increase overall survival rates and facilitate personalized treatment strategies. Thus, studying patients with LM from SBA within the large cohort of the SEER database holds significant clinical relevance.

Although previous studies have developed models for the frequency and prognosis of LM originating from small bowel malignancies, they have focused primarily on small bowel neuroendocrine tumors (NETs) and gastrointestinal stromal tumors (GISTs), with less research dedicated to the risk and prognostic factors of LM in SBA and their impact on patient survival (19). This study investigated the prevalence, risk factors, and prognostic factors of synchronous LM in a large cohort of SBA patients. The nomogram developed in this research aids in identifying patients at high risk for LM and analyzes prognostic factors for patients with SBA combined with LM, assisting clinicians in targeted screening and crafting personalized treatment plans.

The incidence of synchronous LM in SBA patients is 13.37%, which is comparable to the 15.3% occurrence rate of LM in CRC patients (20). Multivariate logistic regression analysis identified independent risk factors affecting LM in SBA patients. In the LM cohort, a greater proportion of male patients (58.3% vs. 41.6%) in the LM cohort suggested that being male could be a risk factor for LM in SBA patients, although this difference was not significant according to regression analysis, which contrasts with findings in CRC LM patients (21). One possible explanation is the inherently greater incidence of SBA in males. Our study indicated that the prevalence of LM varies according to the primary tumor location within the SBA. LM tended to increase in tumors in the lower part of the small bowel. A potential explanation is that, compared to the duodenum, the lower small bowel has richer lymphatic drainage. This extensive lymphatic network could facilitate the spread of cancer cells to the mesenteric lymph nodes, which then disseminate to the liver via systemic circulation (22, 23). Additionally, the blood from the lower segments of the small bowel directly drains into the portal vein system, delivering all absorbed substances directly to the liver. This “first-pass effect” to the liver provides a direct pathway for metastatic cells to colonize the liver (24). The expression of certain cell adhesion molecules (such as integrins and E-cadherin) and growth factors (such as transforming growth factor-beta (TGF-β) and vascular endothelial growth factor (VEGF)), which are involved in tumor invasion and metastasis, may differ between tumors in the lower small bowel and the duodenum, affecting their metastatic potential (25–27). However, this complex issue requires further investigation.

Furthermore, the T stage and N stage also impact the occurrence of LM in SBA, with higher stages showing a greater propensity for LM. Larger tumors and more involved lymph nodes are associated with an increased likelihood of lymphatic, hematogenous, and serosal dissemination (28). The primary mode of treatment for SBA patients is surgical intervention. Missing surgical opportunities often mean that the tumor is left undisturbed, allowing deeper invasion into the intestinal wall and adjacent structures, leading to advanced disease stages where the risk of LM is heightened. Our study underscores the critical role of timely surgical intervention in preventing such outcomes. Fewer regional lymph node dissections increase the chance of LM in SBA patients. Incomplete lymph node dissection may fail to remove all lymph nodes affected by micrometastases, thus allowing cancer cells to spread to the liver via lymphatic and hematogenous routes (29). Moreover, in our study, SBA patients who received chemotherapy were more likely to develop LM, primarily because patients with LM are more in need of chemotherapy rather than chemotherapy itself, leading to LM. SBA is aggressive, and systemic treatments capable of preventing the spread of cancer cells are lacking. Chemotherapy plays a crucial role in targeting microscopic lesions that may not be visible or detectable during diagnosis or surgery (30, 31).

The identification of prognostic factors is crucial for guiding personalized treatment and improving survival rates in patients with LM originating from SBA. In this study, Cox survival regression analysis identified five prognostic factors for patients with SBA-LM. Advanced age is a significant prognostic factor for cancer outcomes. Compared to patients under 50 years of age, those aged 70 years and older had a significantly increased risk of death, consistent with previous studies (32). The potential mechanisms underlying this correlation may involve age-related factors such as decreased immune responses and increased levels of chronic inflammation, which could impact the survival of SBA patients (33, 34). The primary tumor location is also a critical factor for patients with SBA LM. In this study population, more than half of the primary sites of SBAs were in the duodenum (52.4%). The incidence of SBA has been increasing annually, likely due to an increase in the incidence of duodenal cancer (35, 36). This study indicated that, compared to the jejunum, a duodenal location is a negative survival factor for patients, which is consistent with prior research (37). This may be explained by SBA patients whose duodenal locations often present at later stages, which is associated with delayed diagnoses and lower rates of tumor-related surgery (38, 39). Additionally, duodenal cancers are more prone to invade nearby structures such as the pancreas, bile ducts, and mesenteric vessels. Parts of the descending and horizontal duodenum are retroperitoneal, making lymph nodes that are invaded more likely to spread posteriorly, increasing the difficulty of dissection and leading to poorer prognosis (40).

Our study highlights that both radical and palliative surgery, as well as the extent of regional lymph node dissection, have significant impacts on survival outcomes in patients with LM from SBA. Radical surgery aims to completely remove the primary tumor and any resectable metastatic disease, potentially increasing survival rates and achieving a disease-free state. In patients with LM, radical liver resection can be curative if all tumor tissues are removed. Research shows that patients with localized LM who undergo complete resection have significantly greater survival rates than those who do not undergo surgery (41). Palliative surgery is used to alleviate symptoms and prevent complications associated with advanced tumors, such as intestinal obstruction, bleeding, or pain (42). By relieving these symptoms, palliative surgery can improve quality of life and indirectly increase survival rates. Palliative interventions help maintain the patient's nutritional status and overall health, which are crucial for tolerating further treatments such as chemotherapy. Increasing the number of regional lymph nodes dissected can provide more accurate staging and prognostic information, which is essential for planning further treatment. Removing lymph nodes that may contain micrometastases could also reduce the tumor burden and decrease the chances of recurrence (43, 44). The extent of lymph node involvement is a recognized prognostic factor; therefore, thorough lymph node dissection and analysis contribute to better prognostication for patients with LM from SBA.

Moreover, many past studies have demonstrated that adjuvant chemotherapy can significantly improve OS and disease-free survival (DFS) (45, 46). In our study, 69.2% of SBA patients with LM received chemotherapy, and those treated with chemotherapy had a significantly reduced risk of death (HR = 0.46, 95% CI: 0.35–0.61, *P* < 0.001), indicating that chemotherapy plays a positive role in improving patient prognosis. A previous multicenter study of chemotherapy in patients with advanced SBA suggested that FOLFOX may be the most effective platinum-based chemotherapy regimen (12). Unfortunately, the SEER database does not provide information on specific chemotherapy regimens or drug choices, precluding subgroup analyses. We look forward to future updates to the database that may provide this information, allowing for more detailed assessments of the impact of chemotherapy on SBA patients with LM.

However, our study also has several limitations. First, it utilizes information from the SEER database for statistical analysis, and as a retrospective study, it inherently carries biases, necessitating future validation through prospective research. Additionally, since the SEER database only records synchronous LM and does not account for patients who develop LM later in their disease course, the actual overall incidence of LM may be underestimated. Second, we excluded patients with incomplete information, which could have led to selection bias. Furthermore, our study did not include several critical factors, such as tumor markers, body mass index (BMI), or genetic mutation status. These factors are missing from the SEER database and could be relevant to LM and the prognosis of SBA patients. Finally, the nomogram model was constructed using data from only this database, and it remains necessary to evaluate the model's accuracy through external validation in different populations in the future.

## 5 Conclusion

In summary, our study identified several risk factors associated with the occurrence and prognosis of LM in patients with SBA. Based on these factors, a nomogram was developed to predict the risk and prognosis of synchronous LM in SBA patients, showing good discriminative ability and calibration. This nomogram may assist clinicians in predicting individual patient risk and offering improved treatment recommendations. However, future research will require more multicenter external validations to make the results more convincing and directive.

## Data Availability

The raw data supporting the conclusions of this article will be made available by the authors, without undue reservation.
